# A POTENTIAL ZOONOTIC PARASITE: *CRYPTOSPORIDIUM PARVUM* TRANSMISSION IN RATS, PIGS AND HUMANS IN WEST LOMBOK, INDONESIA

**DOI:** 10.21010/ajid.v15i2.8

**Published:** 2021-03-18

**Authors:** Ersandhi Resnhaleksmana, Mahardika Agus Wijayanti, Wayan Tunas Artama

**Affiliations:** 1 Department of Medical Laboratory Technology, Politeknik Kesehatan Mataram, Mataram, Indonesia.Doctoral Program, Faculty of Medicine, Public Health and Nursing, Universitas Gadjah Mada, Yogyakarta, Indonesia; 2 Department of Parasitology, Faculty of Medicine, Public Health and Nursing, Universitas Gadjah Mada, Yogyakarta 55281, Indonesia; 3 Centre for Tropical Medicine, Faculty of Medicine, Public Health and Nursing, Universitas Gadjah Mada, Yogyakarta 55281, Indonesia; 4 Department of Biochemistry, Faculty of Veterinary Medicine, Universitas Gadjah Mada, Yogyakarta, Indonesia; 5 One Health/Ecohealth Resource Center, Universitas Gadjah Mada, Yogyakarta, Indonesia; 6 Department of Medical Laboratory Technology, Politeknik Kesehatan Mataram, Mataram, Indonesia

**Keywords:** Zoonotic parasite, *Cryptosporidium*, Rats, Pigs and Humans

## Abstract

**Background:**

*Cryptosporidium* is a neglected zoonotic disease, but with the expansion of the human community into the animal environment, its incidence is increasing. Animals such as rats and pigs can act as intermediate hosts and transmit *Cryptosporidium* to humans due to their proximity. Transmission occurs due to the ability of *Cryptosporidium* to survive in any new host. The research aimed to identify and describe the transmission of *Cryptosporidium* from animals to humans.

**Materials and Methods:**

This research was a cross sectional study and samples were collected from 84 rats caught in residential areas, 205 pigs, and 438 humans in West Lombok. Fecal samples were examined using polymerase chain reaction (PCR) and sequencing to isolate the presence of *Cryptosporidium*, and identify the genetic similarity of the parasites found in rats and pigs with those that infect humans.

**Results:**

The PCR results found *Cryptosporidium parvum* in 4.76% (4/84) in rats; 6.34% 13/205) in pigs; and 0.91% (4/438) in humans. The sequencing results showed genetic kinship of *C. parvum* in rats, pigs, and humans. Based on sequence confirmation from Gene Banks and edited using ClustalW with MEGA X software, there are genetic similarities between *Cryptosporidium* isolates from West Lombok and *C. suis* isolates of cattle from Uganda and *C. suis* isolates of pigs from Slovakia.

**Conclusion:**

There are genetic similarities of *Cryptosporidium* in animals and humans, requiring that the Public Health programs in those contaminated areas must receive priority attention to prevent further transmission of these potentially fatal parasites.

## Introduction

Zoonotic diseases are increasing especially in developing countries and are becoming neglected diseases. Survey results from 1,407 pathogens in humans showed 58% of emerging infectious diseases and 75% of emerging infectious diseases are zoonotic diseases. An estimated 75 percent of new infectious diseases are zoonotic in origin, directly resulting from human and animal interactions (Woolhouse and Sequeria, 2005; Austin, 2021). Factors contributing to the increase of these diseases are as a result of an increase in population and human activities that have changed the forest environment to the human environment. Natural habitats that have changed their functions as agricultural land, plantations, and shelter, cause humans to live side by side with animals. Rats are reservoirs of infectious diseases because of their habitat and habit of looking for food in dirty places so that the diseases they carry can harm humans. Rat-based diseases began to increase by changes in habitat for animals and their closer proximity to the human environment (Thiermann, 2004; Woolhouse and Sequeria, 2005; Morand, 2015; Sun *et al.*, 2018).

Rats can spread and transmit various infectious diseases to humans and other animals. Rats can carry 61 types of infectious diseases, including 20 types of viruses, 19 types of bacteria, and 22 types of parasites, including *Cryptosporidium spp*. The spread of parasitic zoonotic diseases from rats in the human environment needs to be carefully investigated for the source of parasitic zoonotic transmission. The proximity of rats to the human environment can be a risk factor for transmission of parasites from rats to humans and animals (Perec-Matysiak *et al.*, 2015; Zahedi *et al.*, 2016; Azzam KM, 2017; Krijger, 2020; ).

The presence of poorly organized pig farms and cattle farms has led to the rapid spread of zoonotic parasites. Pollution of soil, water and air around human housing by zoonotic parasites is a result of unsafe animal rearing. Research reports show that farming in a residential environment increases the incidence of diseases in animals and has the potential to spread zoonotic diseases (Mosites *et al.*, 2016). Baqer *et al*. (2018) showed contamination by Cryptosporidium oocysts in a river adjacent to a cattle farm in Baghdad.

*Cryptosporidium parvum* is a zoonotic parasite that can cause gastrointestinal disorders with symptoms such as diarrhea. The incidence of diarrhea can increase morbidity and mortality rates especially in children and is a cause of death of four million lives in developing countries each year (Badry *et al.*, 2014; Verkerke *et al.*, 2014; Dupont, 2016; Yee *et al.*, 2018). Prolonged infection results in dehydration and weight loss. This situation can be severe in children or people with low immunity. Pathological conditions that are caused by these parasitic infections include epithelium damage in the from of villious atrophy, mitochondrial changes and increased lysosomal activity in infected cells (Ridley, 2012; Bogitsh *et al.*, 2013).

*Cryptosporidium* in humans is a recent case that often occurs in areas with poor hygiene, usually involving reports of contact between humans and rats causing the migration of *Cryptosporidium* to humans. Humans are often infected with *C. parvum* and *C. hominis*; cattle with *C. parvum*, *C. bovis*, *C. ryanae*, and *C. andersoni*; while sheep and goats are infected with *C. parvum*, *C. ubiquitum*, and *C. xiaoi*. Most of the species in these animals are also found in humans. So far, more than 20 species of *Cryptosporidium* have been identified in humans. Sequencing methods using the small subunit rRNA gene (SSU rRNA) showed the presence of *C. parvum* and *C. muris* in rats in China and detected *C. parvum* isolate 11dA15G1 identified using the gp60 gene which can infect humans (Zhao *et al.*, 2015; Beser *et al.*, 2020).

The research was conducted on the island of Lombok, where the pigs are often free to find their food around the house or are given leftovers. The poor pig farming practices result in the transmission of parasites between animals and humans. Zoonotic transmissions on the island of Lombok have never been reported. This research concentrated on the transmission of *Cryptosporidium* zoonotic diseases from animals to humans in West Lombok, Indonesia.

## Materials and Methods

### Study design.

The study design was a comparative cross-sectional research. The duration of the study was six months (January, 2019 to June, 2019).

### Samples

The location of fecal sampling is based on the area that has pig farms. Residents who previously volunteered to fill out the informed consent were the sources of the research samples. Stool samples were collected from 84 rats caught and sacrificed from residential areas, 205 pigs, and 438 humans. Samples were taken in West Lombok, Indonesia at 191 locations. The freshness of the stool sample was maintained by the addition of a 5% potassium bichromate preservative.

### Ethical Approval

This study was approved by the Medical and Health Research Ethics Committee of the Faculty of Medicine, Public Health and Nursing, Universitas Gadjah Mada-Dr. Sardjito General Hospital, with approval number: KE/FK/1222/EC/2018, dated 21 November 2018.

### Informed Consent

Informed consent was obtained from all individual participants included in this study.

### Data collection

Interviews method and laboratory examinations of *Cryptosporidium* DNA found in humans, pigs, and mice by PCR and sequencing methods (Munshi, 2012) were employed to gather data for this study.

### Laboratory Methods

###  DNA Extraction

Isolation of DNA was done using the procedures from QIAamp, Fast DNA Stool Mini Kit (Qiagen, German). Stool sample of 180-220 mg was inserted in a 2 ml tube with 1 ml InhibitEX Buffer, and vortexed until the sample was homogeneous. Samples were lysed using a mini Beadbeater for 5 minutes, and continued with the “freeze-Thaw” process, which was incubated at -80^O^C for 5 minutes and incubated 60^O^C in a water bath for 5 minutes (four times). To separate pellets, the sample was centrifuged for 1 minute at 10,000 rpm. 600 µL supernatant was then placed in a pipette, and 25 µL Protein K and 600 µL AL buffer was added, then vortexed for 15 seconds, and incubated 10 minutes at 70^O^C. Next, 600 µL of 96-100% ethanol was added to the lysate and vortexed. Lysate was then put in a spin column, and centrifuged at 10,000 rpm for 1 minute, then the filtrate was removed. Next, 500 µL AW1 buffer was added into the spin column with a new collection tube, centrifuged at 10,000 rpm for 1 minute, and the filtrate removed. Next, 500 µL Buffer AW 2 was added in a new collection tube, and centrifuged at 10,000 rpm for 3 minutes. Then, the spin column was removed from the collection tube then put in a new collection tube, and centrifuged at 10,000 rpm for 3 minutes. Next, the spin column was transferred to the 1.5 ml tube, and 100 - 200 µL buffer ATE was added to the spin column and incubated for 1 minute, then centrifuged at 10,000 rpm for 1 minute. Finally, the spin column was discarded, and the tube containing DNA was stored at -20^O^C.

### PCR Amplification and Detection

Polymerase chain reaction (PCR) used the Bioline mix, with 1 µL DNA template, 7 µL ultrapure water and 10 µL master mix, with 1 µL primers. The primers used were: *Cryptosporidium parvum* gene 18S rRNA, F: 5’-TAAACGGTAGGGTATTGGCCT-3’; R: 5’-CAGACTTGCCCTCCAATTGATA-3 ’. The PCR conditions used were 35 cycles, with an initial activation temperature of 95^O^C for 5 minutes, denaturation temperature of 95^O^C for 30 seconds, annealing temperature of 59^O^C for 45 seconds, an extension temperature of 72^O^C for 3 minutes, and a final extension temperature of 72^O^C for 10 minutes. The final results were examined using electrophoresis at 2% agarose, for presence of *Cryptosporidium parvum* at 240bp (Zebardast *et al.*, 2016).

### Sequencing

Sequencing used the Applied Biosystem 3500 Genetic Analyzer 2500 tool with the Bigdye Terminator kit. DNA *Cryptosporidium* sequences from rats, pigs and human isolates were analyzed using the online BLAST program (NCBI), while sequences from Gene Banks that have genetic similarities with *Cryptosporidium* isolates from Lombok isolates were edited using ClustalW with MEGA X software. Phylogenetic trees were arranged based on neighbor-joining (Kumar *et al.*, 2018).

## Results

**Figure1:**
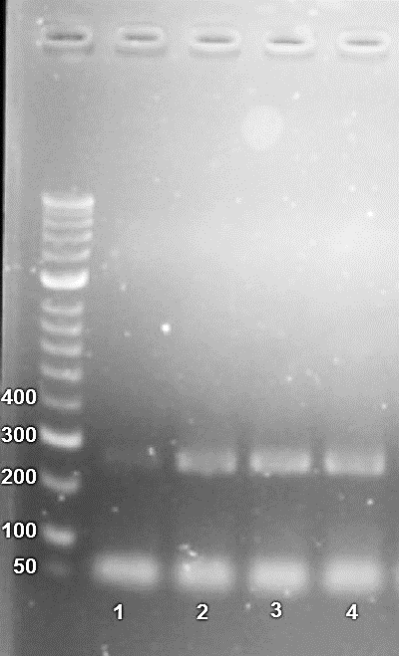
PCR results of *Cryptosporidium parvum* isolates from West Lombok, electrophoresis results in 2% agarose with FloroSafe stain, and HyperLadder Bioline, using 18S rRNA gene showed *Cryptosporidium parvum* 240 bp, No.1. Negative; No.2. Rats; No.3. Pig; and No.4. Human.

PCR results showed *Cryptosporidium* infection in rats 4.76% (4/84), pigs 6.34% (13/205) and humans 0.91% (4/438). Electrophoresis results on agarose 2% are shown in Figure 1.

**Figure 2:**
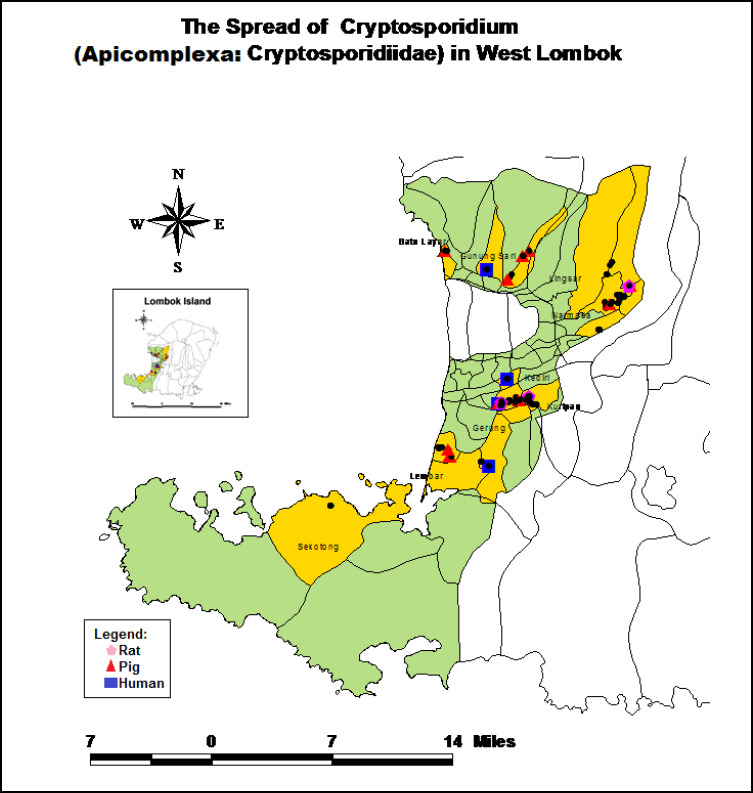
*Cryptosporidium parvum* from rats, pigs, and humans in West Lombok, *C. parvum* are found in the districts of Narmada, Kediri, and Gerung. *C. parvum* also was identified in Gunungsari and Lembar. The infection rate in rats was 4.76% (4/84), in pigs 6.34% (13/205) and in humans 0.91% (4/438).

*Cryptosporidium parvum* from isolates of rats, pigs and humans in Lombok was spread in several districts, and the distribution is shown in Figure 2.

Genetic similarity of *Cryptosporidium parvum* sequences of rat, pig and human isolates with DNA gene sequences from the NCBI Bank using the BLAST program online on the website (Http: //www.ncbi.nlm. nih.gov). DNA sequences of *Cryptosporidium parvum* isolates of rats, pigs, and humans based on the 18s rRNA gene are as follows:

*Cryptosporidium* in Rats

GTAGGGTATTGGCCTACCGTGGCAGTGACGGGTAACGGGGAATTAGGGTTCGATTCCGGAGAGGGAGCCTGAGAAACGGCTACCACATCTAAGGAAGGCAGCAGGCGCGCAAATTACCCAATGAAAACAGTTTCGAGGTAGTGACGAGAAATAACAATACAGGGCATTTTTTGCTCTGTAATTGGAATGATGGGAATGTAAAACCCTTTCCAGAGTATCAATTGGAGGGCAAGTC

*Cryptosporidium* in Pigs

GGTAGGGTATTGGCCTACCGTGGCAGTGACGGGTAACGGGGAATTAGGGTTCGATTCCGGAGAGGGAGCCTGAGAAACGGCTACCACATCTAAGGAAGGCAGCAGGCGCGCAAATTACCCAATGAAAACAGTTTCGAGGTAGTGACGAGAAATAACAATACAGGGCATTTTTTGCTCTGTAATGGAAATGATGGGTATAAGAAGGCCTTTCCAGAGTATCAATTGGAGGGCAAGTCTGGACTAG

*Cryptosporidium* in Humans

TTGGCCTACCGTGGCATTGACGGGTAACGGGGAATTAGGGTTTGATTCCGGAGAGGGAGCCTGAGAAACGGCTACCACATCTAAGGAAGGCAGCAGGCGCGCAAATTACGCAATATCAACGAAAGGGCGATAGGATCTAAACGGTCGGGTGTTAACCTTCTTAACAAGTATCAATTGGAGGGCAAGTCTGGAAC

BLAST of the DNA sequences of *Cryptosporidium parvum* isolates of rats, pigs, and human genes are aligned with the sequences of the Gene Bank shown in the Table 1.

**Table 1:** Blast *Cryptosporidium* DNA sequences of gene Bank based on 18s rRNA.

Description	Max. Score	Total Score	Query cover	E value	Per. Ident	Accession
*C. parvum,* obi24	303	303	100%	8e-83	90.38%	FJ796279.1

*C. parvum,* obi21	303	303	100%	8e-83	90.38%	FJ796279.1

*C. parvum,* 04IR(NG)	303	303	100%	8e-83	90.38%	AB441688.1

*C. hominis*, 24937	195	249	75%	2e-50	97.37%	MG952704.1

*C. hominis,* 28-9	195	249	75%	2e-50	97.37%	MK270514.1

*C. hominis, Human*-IQ7	195	249	75%	2e-50	97.37%	MK886605.1

*C. suis*, B8	305	305	100%	1e-85	90.38%	MG132078.1

*C. suis*, HK107C	267	267	97%	6e-73	92.19%	MK301308.1

*C. suis*, Swec705	305	305	100%	7e-83	90.38%	MH187877.1

C. suis	305	305	100%	7e-83	90.38%	KP704556.1

C. suis, B14	305	305	100%	7e-83	90.38%	KT223028.1

C. suis, CWQ4	305	305	100%	7e-83	90.38%	KJ790239.1

**Figure 3:**
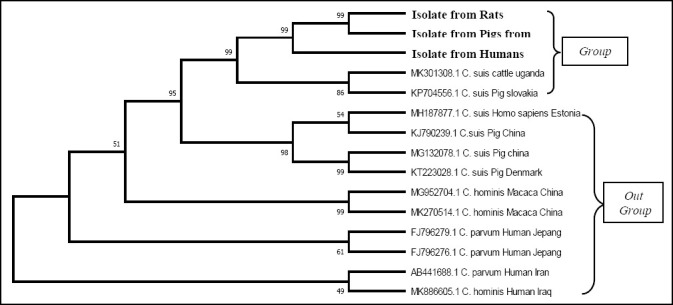
Phylogenetic tree of *Cryptosporidium parvum* isolates of rats, pigs and humans from the alignment with gene bank sequences based on the 18s rRNA gene. *Cryptosporidium parvum* isolates of Rats, pigs, and humans in West Lombok are one group with isolate MK301308.1 *Cryptosporidium suis*, Cattle, Uganda and isolate KP704556.1 *Cryptosporidium suis*, pigs, Slovakia. *Cryptosporidium suis* isolate MH187877.1 Homo sapiens, Estonia; KJ790239.1, MG132078.1 pig, Chinese; KT223028.1 pig, Denmark; *Cryptosporidium hominis* isolate MG952704.1, MK270514.1 Macaca, China; MK886605.1 Human, Iraq; *Cryptosporidium parvum* isolate MG952704.1, MK270514.1 Macaca China; MK886605.1 Human Iraq; *Cryptosporidium parvum* isolate FJ 796279.1 human, Japanese; and AB441688.1 Human, Iran is an outgroup.

Phylogenetic *Cryptosporidium parvum* in rats, pigs, and humans in West Lombok, and DNA sequences from Gene Banks are used to construct phylogenetic trees. Phylogenetic tree is composed of the number Acc: FJ796279.1 *C. parvum*, Human, Japan; FJ796276.1 *C. parvum*, Human, Japan; AB441688.1 *C. parvum*, Human, Iran; MG952704.1 *C. hominis*, Macaca, Chinese; MK270514.1 *C. hominis*, Macaca, Chinese; MK886605.1 *C. hominis*, Human, Iraq; MH187877.1 *C. suis*, Homo sapiens, Estonia; MK301308.1 *C. suis*, cattle, Uganda; MG132078.1 *C. suis* Pig, china; KP704556.1 *C. suis*, Pig, Slovakia; KT223028.1 *C. suis*, Pig, Denmark; KJ790239.1*C.suis*, Pig, China. Genetic kinship analysis using sequence alignment is based on Gene Bank. Bootstrap tree consensus with the conclusion of 1000 replications and parasitic distance evolution was calculated based on the “Kimura 2” method. Phylogenetic analysis used “MEGA X” software (Kimura, 1980; Felsenstein, 1985; Saitou and Nei, 1987). Figure 3 shows the results.

Genetic kinship of *Cryptosporidium* isolates of rats, pigs and humans, confirmed by the pairwise distances calculation to the DNA sequence of the gene Bank (NCBI), with genetic distances are shown in Table 2.

**Table 2:** Genetic distance of *Cryptosporidium parvum* DNA sequences of rat, pig and human isolates from DNA sequences from bank genes.

No	Sample	Genetic distance		

		Rats	Pigs	Humans
1	**Isolate from Rats**			
2	**Isolate from Pigs**	0.0104		
3	**Isolate from Humans**	0.2534	0.3166	
4	FJ796279.1 *C. parvum* *Human* Japan	0.1003	0.1462	0.3112
5	FJ796276.1 *C. parvum* *Human* Japan	0.1003	0.1462	0.3112
6	AB441688.1 *C. parvum* *Human* Iran	0.1003	0.1462	0.3112
7	MG952704.1 *C. hominis Macaca* China	0.1003	0.1462	0.3112
8	MK270514.1 *C. hominis Macaca* China	0.1003	0.1462	0.3112
9	MK886605.1 *C. hominis Human* Iraq	0.1003	0.1462	0.3112
10	MH187877.1 *C. suis Homo sapiens* Estonia	0.1056	0.1506	0.3030
11	MK301308.1 *C. suis cattle* Uganda	0.1056	0.1377	0.2710
12	MG132078.1 *C. suis Pig* China	0.1056	0.1506	0.3030
13	KP704556.1 *C. suis Pig* Slovakia	0.1056	0.1506	0.3030
14	KT223028.1 *C. suis Pig* Denmark	0.1056	0.1506	0.3030
15	KJ790239.1 *C. suis Pig* China	0.1056	0.1506	0.3030

The DNA sequence of *Cryptosporidium parvum* isolates of rats, pigs, and humans has a tight genetic range, which is: 0.0104-03112 from the Gene Bank sequence. The sequences from Gene Bank are: *C. parvum* human isolates from Japan, Iran; *C. hominis* Macaca isolate from China, human from Iraq, Estonian *Homo sapiens*; and *C. suis* pig isolates from China, Slovakia, Denmark.

## Discussion

*Cryptosporidium* identified in rats, pigs and humans in West Lombok was 4.76% (4/84), 6.34% (13/205) and 0.9% (4/438), respectively, by PCR at 240bp. *Cryptosporidium* was also identified in other countries using PCR analysis. El-Bakri *et al*. (2018) identified twenty-six individuals (19.4%) who were positive for *Cryptosporidium* among asymptomatic healthy expatriate workers in Sharjah, United Arab Emirates. Meanwhile, Elmatrawy *et al*. (2017) identified *Cryptosporidium* spp. in children with diarrhea in Egypt 6% (9/150); while Bodager *et al*. (2015), identified *Cryptosporidium* with gene signatures from rat 2.08% (1/48), pig 1.65% (3/17) and human 0.83% from (1/120) human samples in Ranomafana National Park, Madagascar.

The similarity of DNA sequences of *Cryptosporidium parvum* isolates of rats, pigs and humans are shown by the Mega X program. These results identify genetic similarities of *C. parvum* that infect rats, pigs, and humans. Genetic similarity is related to the emergence of *C. parvum* zoonotic infections from rats and pigs to humans. Molecular analysis of the *Cryptosporidium parvum* uses the 18S rRNA gene because it is a reference gene that is often used as an internal control in the analysis of gene expression. Reference genes are genes whose expression is stable, not induced by certain treatments, abundant in all tissues, and follow the stages of the eukaryotic development. The 18S rRNA gene encodes an 18S ribosomal RNA gene, as a constituent of the eukaryotic small subunit ribosome in the process of recognition and hybridization of mRNA that is translated in the ribosome (Thellin *et al.*, 1999; Dresios *et al.*, 2006) The 18S rRNA gene is also used to detect *Cryptosporidium* from porcupine isolates in the UK, which was detected in 8% (9/111). The 18S rRNA gene also showed good results for detecting *Cryptosporidium* in pigs in Australia with the Next Generation Sequencing (NGS) method (Paparini *et al.*, 2015; Sangster *et al.*, 2016).

*Cryptosporidium parvum* isolates of rats, pigs and humans have a genetic similarity to DNA sequences from NCBI (the max score. 303, total score 303, query cover 97%, E-value 53-73 and per. ident 92.71%). The taxonomic results of the gene *C. parvum* have the highest scores than other species. Phylogenetic tree kinship analysis used the neighbor-joining method with bootstrap 1000x. The genetic relationship between *C. parvum* isolates of rats and pigs in West Lombok is monophyletic and *C. parvum* isolates of humans in West Lombok are synapomorphic with isolates of rats and pigs. Pairwise distance calculation between *Cryptosporidium* isolates of rats, pigs, and humans in West Lombok was 0.000-0.3170, indicating there is a close genetic relationship between *C. parvum* in rats, pigs, and humans in West Lombok.

*C. parvum* identified in humans in West Lombok comes from rats and pigs. *C. parvum* found in West Lombok is a zoonotic parasite, and there has been a transmission of *Cryptosporidium* infection from rats and pigs to humans. The presence of *C. parvum* in rats and pigs is a new source of transmission from *C. parvum* in West Lombok. The results of research on zoonotic *Cryptosporidium* were also reported by Deng *et al*. (2020), while *C. parvum* was identified in 8.6% (27/314) from the phylogenetic analysis of red squirrel pets sold in Sichuan, China. The presence of *C. parvum* in red squirrels is suspected as the source of transmission of *C. parvum* to humans and causes diarrhea. Phylogenetic analysis of *C. parvum* isolates of rats, pigs and humans are located in a genetic kinship group with isolates KP704556.1 *C. suis* pig, Slovakia and MK301308.1 *C. suis*, cattle, Uganda (Gordon, 2003).

Pairwise distance calculation analysis showed the genetic relationship between *Cryptosporidium* rats, pigs, and humans in West Lombok with *Cryptosporidium* Gene Bank isolates in Slovakia with genetic distance 0.1060-0.3030 and pigs in Uganda with genetic distance 0.1060-0.2710. Pairwise distance calculation analysis is used to determine the transition substitution and transversions through the many different nucleotides per base pair. Species with genetic distance that are getting closer have a strong genetic relationship (Dharmayanti, 2018).

*Cryptosporidium parvum* infection in West Lombok may be derived from rats and pigs, while the results of alignment with Gene Bank isolates showed a genetic relationship with *C. parvum* isolates of rats and pigs. These results concur with the study by Zou *et al*. (2017), identifying *Cryptosporidium* in pig farms in China using the 18S rRNA gene finding *Cryptosporidium* infection of 8% - 23%, which can act as a zoonotic source to humans. Utsi et al. (2016) found that a cryptosporidiosis outbreak in America was caused by visitors infected with *Cryptosporidium*, after returning from a petting farm.

Volunteers who have provided stools samples in this study had a house close to the pig farm. The pigs’ fecal litter was strewn in the yard and sometimes thrown into the garden or the river. The source of pigs fodder comes from recycling food scraps from residents.

The presence of rats around cages and houses, and the activity of rats moving from pigpens to poeple’s homes can carry and transmit diseases from rats to humans or to other animals. *C. parvum* identified in rats, pigs, and humans in West Lombok shows that there is a connection between *Cryptosporidium* transmission from rats, pigs, and humans. *C. parvum* which infects rats, pigs and humans is a zoonotic intestinal parasite, and this parasite may contribute to the incidence of diarrhea in West Lombok. The same case has occurred in Korea, where *C. parvum* was identified in 0.37% (32/8,571) of hospital diarrhea samples (Ma *et al.*, 2019). *Cryptosporidium* zoonosis has also occurred in Madagascar and infects rats, pigs and humans around the national park (Bodager *et al.*, 2015).

*Cryptosporidium parvum* infection in West Lombok can originate from rats and pigs. Zoonoses occur due to risk factors for environmental hygiene, raising pigs, and the presence of rats. This is in accordance with the opinion of Innes *et al*. (2020), that the presence of pigs, cows, horses and other wild animals such as rats around residential areas presents a high risk of polluting the environment by animal feces containing *Crytptosporidium* oocytes. Contamination of the environment by animal feces facilitates transmission of *Cryptosporidium parvum* to humans.

Feng *et al*. (2018) identified transmission between *Cryptosporidium* from goats to cattle that can even infect humans. Most *Cryptosporidium* species and genotypes have a host specificity so that one to four of the *Cryptosporidium* species can be found in one host (Widmer *et al.*, 2020). The ability of the parasite to adapt within the new host and geographical pressure causes the formation of unique subtypes and phenotypic properties, especially those found in humans (*C. parvum* and *C. hominis*) (Feng *et al.*, 2018). The intensity of transmission, genetic diversity, and genetic recombination form the genetic tree structure of *Cryptosporidium*. Molecular research on *Cryptosporidium* spp. can help increase our understanding of various patterns of transmission of *Cryptosporidium* to new hosts (Thompson, 2013).

## Conclusions

There are genetic similarities of *Cryptosporidium spp*. that infect rats, pigs and humans in the West Lombok Regency, West Nusa Tenggara Province. This allows the transmission of the parasitic zoonoses *Cryptosporidium* which infect rats, pigs and humans in the West Lombok Regency, West Nusa Tenggara Province based on parasitic genetic kinship. The results of this study require that Public Health programs in contaminated areas receive priority attention to prevent further transmission of this potentially fatal parasite. More research is needed to see what risk factors contribute to zoonoses.

**Abbreviations:**
(SSU rRNA): Small Subunit rRNA gene
(DNA): Deoxyribonucleic Acid
(RNA): Ribonucleic Acid
(PCR): Polymerase chain reaction
(BLAST): Basic Local Alignment Search Tool
(NCBI): National Center for Biotechnology Information
(MEGA X): Molecular Evolutionary Genetics Analysis X
(NGS): Next Generation Sequencing
